# Source Apportionment-Based Assessment of Ecological and Health Risk for Potentially Toxic Elements in the Soil Around a Uranium Mine

**DOI:** 10.3390/toxics14090748

**Published:** 2026-08-25

**Authors:** Min Fan, Haiyan Liu, Zeqiang Chen, Zhao Chen, Zhen Wang, Binyu Lu, Narsimha Adimalla

**Affiliations:** 1 National Key Laboratory of Uranium Resources Exploration—Mining and Nuclear Remote Sensing, East China University of Technology, 418 Guanglan Road, Jingkai District, Nanchang 330013, China; fan_min09@163.com (M.F.); chen_zhao@ecut.edu.cn (Z.C.); 201960197@ecut.edu.cn (B.L.); adimallanarsimha@gmail.com (N.A.); 2 Jiangxi Provincial Key Laboratory of Genesis and Remediation of Groundwater Pollution, School of Water Resources and Environmental Engineering, East China University of Technology, 418 Guanglan Road, Jingkai District, Nanchang 330013, China

**Keywords:** source apportionment, potentially toxic elements, risk assessment, APCS-MLR receptor model, mine water and soil pollution

## Abstract

Uranium is a critical strategic resource. However, uranium mining can cause severe contamination of surrounding soils by potentially toxic elements (PTEs). Conventional approaches that separately perform source apportionment and risk assessment fail to establish a direct linkage between pollution sources and their associated environmental and health risks. Herein, we develop an integrated framework that combines the absolute principal component score–multiple linear regression (APCS-MLR) model, the potential ecological risk index, and Monte Carlo simulation. The established framework was used to try quantifying the potential sources of soil PTEs and their corresponding ecological and human risks in a uranium mining area. The results showed that the concentrations of U, Th, Cd, and Cr significantly exceeded local background levels, with Cd and U exhibiting higher spatial variability than the other PTEs. The APCS-MLR model identified three potential sources of soil PTE contamination: mining activities, mixed anthropogenic-natural, and natural sources. Mining and mixed sources were the dominant contributors to ecological risk, jointly accounting for 88.7% of the total ecological risk, with Cd identified as the primary ecological risk pollutant. The mixed sources also contributed the largest proportions of non-carcinogenic and carcinogenic health risks, accounting for 52.56% and 67.5%, respectively. Furthermore, children were found to face greater health risks than adults due to higher exposure levels. Priority factor analysis indicated that pollution management should continuously monitor Cd derived from mining activities based on statistical inference. Overall, the proposed integrated framework successfully established a quantitative linkage between pollution sources and associated risks, providing a scientific basis for source-specific and zone-specific soil pollution management in uranium mining areas.

## 1. Introduction

Soil is a fundamental component of terrestrial ecosystems, and its environmental quality is closely associated with ecological security, food security, and human health [1,2,3]. Potentially toxic elements (PTEs) are characterized by their toxicity, persistence, and concealed nature in the environment [4]. Once accumulated in soil above certain concentrations, PTEs can impair ecosystem functions and threaten human health, making them a major environmental concern that affects regional sustainable development [5,6,7]. Uranium mining and metallurgical activities, particularly the long-term storage of mine tailings in impoundments, promote the accumulation of PTEs in surrounding soils [8,9,10]. The soils around uranium mines are commonly affected by conventional PTEs, which can result in greater environmental risks and pose more challenges for regional management than single-contaminant pollution [11,12,13].

Following decades of uranium mining, soil environmental pollution has become an issue of increasing concern [14,15]. Previous studies have shown that the concentrations of heavy metals, including Cu, Cr, Zn, Pb and Cd, frequently exceed local soil background values [16]. These PTEs can be transported to surrounding farmland and residential areas through water migration, atmospheric deposition, and surface runoff, where they continuously accumulate in soils. Such contamination not only disrupts nutrient biogeochemical cycling and biodiversity within farmland ecosystems, but also poses potential human risks to local residents through multiple exposure pathways, including the soil-crop-human food chain, dermal contact, inhalation [15,17,18].

To better understand the sources of soil PTEs contamination and evaluate the associated risks, source apportionment and risk assessment techniques have been progressively applied in recent studies [19,20,21]. Indeed, receptor models, particularly positive matrix factorization (PMF) and absolute principal component score–multiple linear regression (APCS-MLR), are widely applied to identify sources of soil PTEs [22,23,24]. For example, source apportionment-based on correlation analysis indicated that PTE contamination in soils surrounding a mine in Guangxi province primarily originated from industrial and mining activities, natural sources, agricultural inputs, atmospheric deposition sources and unidentified sources [25]. In parallel, pollution assessment methods such as the potential ecological risk index and the Nemerow comprehensive pollution index have been extensively employed to evaluate soil contamination. Ecological risk assessments have consistently revealed severe PTE pollution in farmland soils [10,15,26,27]. Furthermore, previous studies suggested that farmlands adjacent to smelters experienced the most severe uranium contamination; whereas, farmlands surrounding tailings dams were predominantly polluted by Cd, Cr, Cu and Pb [15]. Despite these advances, several limitations remain. Firstly, conventional source apportionment techniques mostly focus on identifying the pollution sources and fail to quantify the contributions of individual sources to ecological and human health risks, thereby limiting the effectiveness of targeted pollution control strategies [24]. Secondly, routine risk assessments generally evaluate either conventional heavy metals or radionuclides separately, ignoring the combined pollution effects of these contaminants. Interactions between radionuclides and heavy metals during migration, transformation, bioavailability, and toxicological processes may lead to risk estimates that differ from actual environmental conditions [13,28]. Thirdly, most health risk assessments are based on deterministic exposure parameters and fail to account for uncertainties in variables such as daily intake, contact frequency. Consequently, these approaches cannot adequately characterize the probabilistic nature of health risks [29,30,31].

To address these limitations, recent studies have attempted to integrate source apportionment with the potential ecological risk method. For example, Wu et al. combined principal component analysis with ecological risk assessment to identify pollution sources and key control factors on Hainan Island, demonstrating the effectiveness of this integrated framework in improving the precision of pollution management [27]. Nevertheless, the integration of source apportionment with Monte Carlo into a unified frame-work based on probabilistic health risk assessment and potential health risk assessment, and establishment of a quantitative correlation between pollution sources and ecological/health risks are still relatively rare in soil research in uranium mining areas.

Against this backdrop, the present study selected a representative uranium mining area in southern China to develop an integrated source apportionment-risk assessment framework. The APCS-MLR receptor model was first employed to quantify the primary sources of soil PTEs and their respective contributions. The receptor model mainly reveals the statistical relationship between pollutants and potential sources, while source identification combines geological information and land use characteristics with independent field observations. Subsequently, the potential ecological risk index was applied to evaluate ecological risks associated with combined PTE pollution. Finally, a Monte Carlo simulation-based health risk assessment model was introduced to quantify the probabilistic human health risks associated with different exposure pathways. The specific objectives of this research are to (1) characterize the spatial distributions pattern and potential sources of soil PTEs; (2) assess the ecological and health risks of soil PTEs associated with different pollution sources based on source apportionment; and (3) establish an integrated methodological framework linking sources of soil PTEs to probabilistic risk assessment. The findings of this study provide a scientific basis for identifying priority pollutants, and pollution sources, thereby supporting source-oriented soil remediation and risk management in uranium mining areas.

## 2. Materials and Methods

### 2.1. Study Area

**Figure 1:**
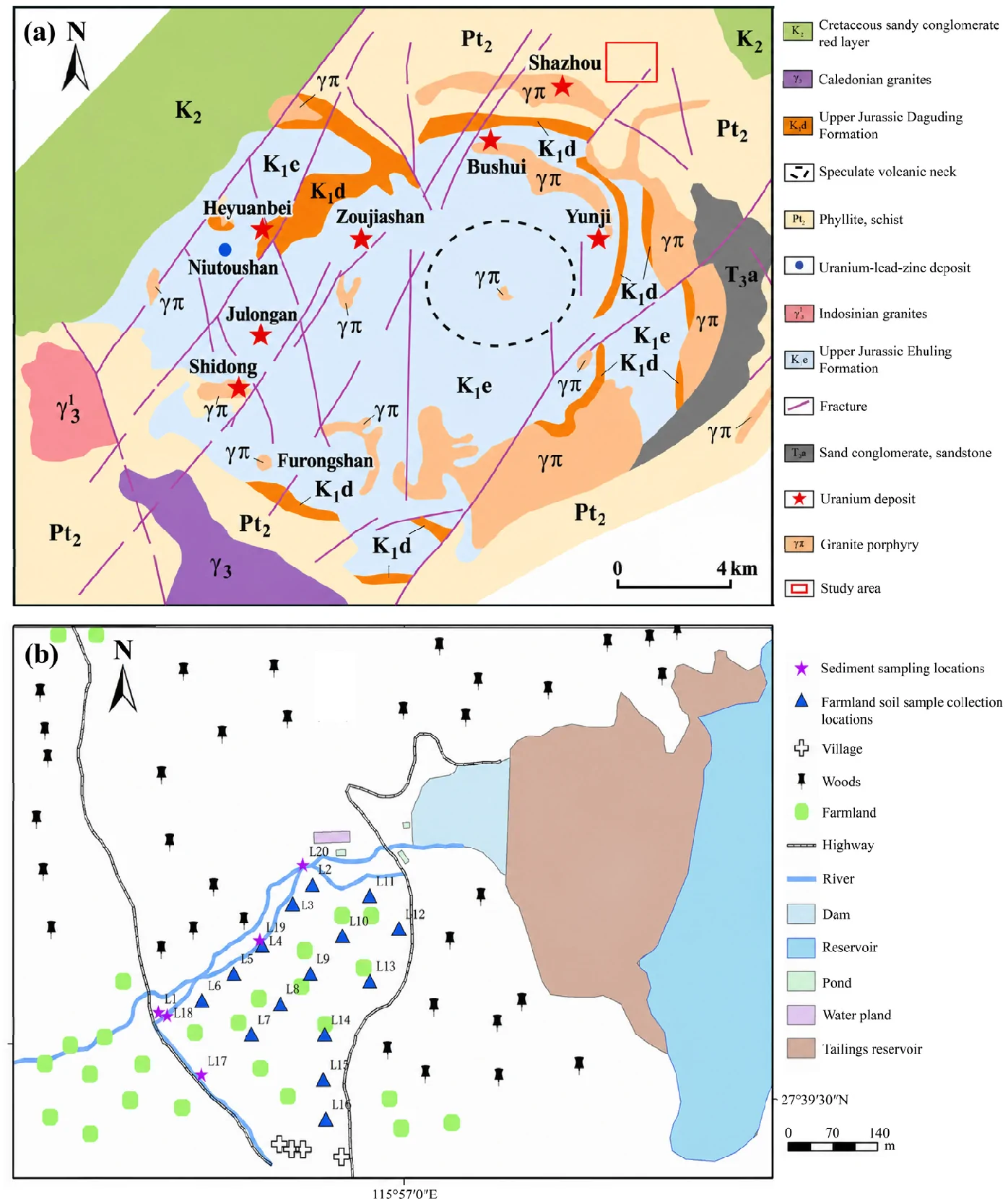
Geological map of the study area (**a**) and sampling locations (**b**).

The uranium mine is located in Jiangxi Province, southern China. The mining area extends approximately 19.3 km from north to south, and 28.5 km from east to west, covering a total area of 320 km^2^. The terrain generally slopes from west to east and exhibits diverse geomorphological features. The study area is characterized by a well-developed drainage system, with dense river networks and numerous tributaries arranged in a rhombic grid pattern, forming a complete and interconnected hydrological drainage network. The stratigraphy of the uranium deposit comprises a basement complex overlying by a cap rock sequence [32,33]. The basement is dominated by early Mesoproterozoic metamorphic rocks, including phyllite and mica quartz schist (Figure 1a). The cap rocks mainly consist of volcanic sequences belonging to the Lower Cretaceous Daguding and Ehuling Formations, which are predominantly composed of volcanic lavas and pyroclastic rocks [34].

The study area has a subtropical humid monsoon climate [35], with a mean annual temperature of 19.4 °C and an average annual precipitation of 1563 mm. Land-use in the surrounding area is dominated by agricultural land and forestland. Farmlands account for approximately 35% of the total area, and are primarily cultivated with rice, rapeseed, and peanuts. Apart from a water treatment plant, no other industrial facilities are located within the research zone. The land use type in the study area is mainly farmland with acidic characteristics. The lithology is mainly composed of silt and clay, belonging to the type of silty clay.

### 2.2. Sample Collection, Pretreatment and Analysis

Soil sampling was conducted from 1 to 5 October 2024. The distribution of the sampling locations is shown in Figure 1b. A total of 37 individual soil samples were collected from the drainage channel at the bottom of the tailings impoundment dam and the adjacent farmlands in the northern part of the uranium mining area. Five sampling locations were arranged along the drainage channel at the dam bottom (L1, L17, L18, L19, L20), where approximately 1 kg soil was collected at 20 cm depth intervals and sealed in labeled polyethylene bags. Fifteen sampling locations were established in the surrounding farmlands. After removing the upper 30 cm of backfill soil, samples were collected from two depth intervals (0–20 cm and 20–40 cm) at each location, which roughly corresponded to the AB transition layer and the Bt viscous deposition layer. At locations L9, L11 and L13, only the 0–20 cm soil layer was sampled because of high soil hardness. All collected soil samples were sealed immediately after collection and transported to the laboratory within 24 h.

Upon arrival at the laboratory, the soil samples were firstly air-dried, after which visible impurities were removed. The samples were subject to grinding and sieving through a 0.074 mm mesh. The sieved samples were then oven-dried to constant weight prior to chemical analysis.

Particle-size distribution (clay, silt, and sand fractions) was analyzed by the pipette sedimentation method. Total organic carbon (TOC) and total nitrogen (TN) were quantified via dry combustion using an Elementar Vario MAX cube elemental analyzer (Elementar, Langenselbold, Germany). Soil pH was measured in a 1:5 (mass/volume) soil–deionized water suspension with a Mettler-Toledo FE28 pH-meter equipped with a composite pH electrode (Mettler-Toledo, Greifensee, Switzerland). All TOC and TN results are reported as mass percentages on an oven-dry soil basis (105 °C), and hygroscopic moisture content of air-dried samples was pre-determined and corrected in all mass-fraction calculations.

Trace element analysis of soil samples was performed in a clean laboratory following the Chinese national standard HJ 781-2016 [36]. Specifically, 0.3 g of each soil sample was weighed into a crucible, and 1 mL of ultrapure water was added to moisten the sample. The crucible was placed on an electric hot plate and preheated to 180 °C under well-ventilated conditions. Subsequently, 5 mL of concentrated hydrochloric acid was added, and the sample was heated until nearly dry. Concentrated hydrochloric acid, hydrofluoric acid and perchloric acid were then added sequentially at a volume ratio of 5:5:3. The samples were further heated at 180 °C on the electric hot plate until the remaining solution was reduced to approximately 2 mL. During heating, the crucible was gently shaken until dense white fumes appeared, after which it was covered and heating continued until the residues turn viscous. After digestion, the crucible was cooled to approximately 40 °C and 2 mL of 2% diluted nitric acid was added to fully dissolved soluble residues. The mixture was cooled to room temperature and quantitatively transferred to a 50 mL volumetric flask. The crucible was rinsed several times with diluted nitric acid, and all rinsing solutions were collected into the flask. The solutions were diluted with 2% nitric acid, and all rinsing solutions were combined in the same flask. The final solution was diluted to volume with 2% nitric acid, thoroughly mixed, and stored prior to instrumental analysis. Elemental concentrations were determined using inductively coupled plasma mass spectrometry (ICP-MS). Before analysis, a series of calibration standards solutions were prepared from certified stock solutions, with concentration ranges covering the expected element concentrations in the soil samples. Three parallel sub-samples were measured simultaneously for each soil sample. The relative standard deviations of the measured PTE concentrations were all below 5%. All analytical results were reported to three significant figures, consistent with the method detection limits.

### 2.3. Source Apportionment Method

The APCS-MLR model quantifies the contribution rates of different pollution sources by establishing a multiple linear regression relationship between the measured concentrations of PTEs in soil and the corresponding absolute principal component scores [37,38,39]. The measured concentrations of all soil PTEs were first standardized to acquire normalized factor scores of zero-concentration samples were then calculated for each PTE. The corresponding formulas are shown in Equations (1) and (2):

(1) $$Z_{\mathrm{ni}}=\frac{C_{\mathrm{ni}}-\bar{C_{i}}}{\delta_{i}}$$

(2) $$Z_{0i}=\frac{C_{0i}-\bar{C_{i}}}{\delta_{i}}$$

where Z_ni_ is standardized concentration of PTE_i_ in the n-th sample; Z_0i_ is standardized value of the zero-concentration sample of PTEi; C_ni_ is concentration of PTE_i_ in the n-th sample (mg/kg); $\bar{C_{i}}$ is average concentration of PTE_i_ (mg/kg); δ_i_ is standard deviation of PTE_i_.

Absolute principal component scores (APCS) were obtained by subtracting the factor scores of zero-concentration samples from normalized factor scores. Using the APCS values as independent variables and the measured concentrations of each PTE as dependent variables, a multiple linear regression equation was established. The contribution rate of each pollution source to soil PTE concentrations was further calculated using the regression coefficients according to Equation (3):

(3) $$C_{j}=\;b_{j0}+\;\sum_{p=1}^{n}b_{\mathrm{pj}}\times \;{\mathrm{APCS}}_{P}$$

where C_j_ is measured concentration of PTE_j_ (mg/kg); APCS_p_ is absolute principal component score of pollution source p; b_j0_ is constant term; b_pj_ is regression coefficient of PTE_j_ against pollution source p; the term bpj × APCS_p_ represents the contribution of source p to C_j_. Certain negative correlations were observed between pollution sources and PTE concentrations. Absolute values of the regression coefficients were used to calculate the source contribution rates.

### 2.4. Source Apportionment-Based Potential Ecological Risk Assessment Method

This method integrates source apportionment with the potential ecological risk index. Based on APCS-MLR results, the contributions of different pollution sources were incorporated into the potential ecological risk model to quantify ecological risks associated with PTEs originating from individual sources [23,40,41]. The calculation formulas are presented in Equations (4) and (5):

(4) $$P_{i}=\frac{C_{i}}{S_{i}}$$

(5) $$\mathrm{RI}=\sum_{i=1}^{n}E_{r}^{i}=\sum_{i=1}^{n}T_{r}^{i}\times P_{f}^{i}=\sum_{i=1}^{n}T_{r}^{i}\times \frac{P_{r}^{i}}{P_{n}^{i}}$$

where P_i_ is single pollution index of element i; C_i_ is measured concentration of element i; Si is the local geochemical background value of element i in soil; $E_{r}^{i}$ is potential ecological risk of PTE i in soil; $P_{f}^{i}$ is single pollution factor of PTE i; $T_{r}^{i}$ is toxic response factor of the i-th PTE (Zn = 1, Ni = 5, Cu = 5, Pb = 5, Cr = 2, Cd = 30).

The potential ecological risk index (RI) is classified into four risk grades: low risk (RI < 150), moderate risk (150 ≤ RI < 300), high risk (300 ≤ RI < 600), and extremely high risk (RI ≥ 600). The assessment criteria for the potential ecological RI are listed in Table 1.

### 2.5. Integration of Source Apportionment and Human Health Risk Assessment

Source apportionment was further integrated with human health risk assessment. Based on the APCS-MLR results, the contributions of different pollution sources were incorporated into human health risk assessment model to quantify health risks associated with PTEs originating from individual pollution sources [42,43]. Specifically, the contribution of each PTE from a single source was calculated based on the source contribution ratio output by the APCS-MLR model. Subsequently, these source specific concentrations were introduced into the US Environmental Protection Agency’s health risk model using parameters related to overall risk assessment (RfD and SF). This method assumed that the health risk contribution of each source was proportional to its contribution to PTE concentration. Therefore, the calculated source specific risks represented the relative risk allocation between pollution sources, rather than independent toxicological effects caused solely by a single source.

The health risk assessment model developed by the United States Environmental Protection Agency (USEPA) is a well-established tool for evaluating site-specific health risks and has been widely applied in environmental health research [44]. Oral ingestion, inhalation, and dermal contact were considered the three dominant exposure pathways through which PTEs may pose risks to human health [44,45]. Human health risks were evaluated by calculating risk indices for these three exposure pathways using Equations (6)–(8):

(6) $${\mathrm{ADD}}_{\mathrm{ing}}=\frac{{\mathrm{EPC}}_{\mathrm{soil}}\times \mathrm{IngR}\times \mathrm{EF}\times \mathrm{ED}}{\mathrm{ABW}\times T_{\mathrm{avg}}}\times 10^{-6}$$

(7) $${\mathrm{ADD}}_{\mathrm{inh}}=\frac{{\mathrm{EPC}}_{\mathrm{soil}}\times {\mathrm{InhR}}_{\mathrm{soil}}\times \mathrm{EF}\times \mathrm{ED}}{\mathrm{PEF}\times \mathrm{ABW}\times T_{\mathrm{avg}}}\times 10^{-6}$$

(8) $${\mathrm{ADD}}_{\mathrm{der}}=\frac{{\mathrm{EPC}}_{\mathrm{soil}}\times \mathrm{ESSA}\times \mathrm{SAF}\times \mathrm{DAF}\times \mathrm{EF}\times \mathrm{ED}}{\mathrm{ABW}\times T_{\mathrm{avg}}}\times 10^{-6}$$

where ADD_ing_ is daily absorbed dose of PTEs via oral ingestion; ADD_inh_ is daily absorbed dose via inhalation; ADD_der_ is daily absorbed dose via dermal contact; EPC_soil_ is measured PTE concentration in soil; IngR is soil ingestion rate; InhR_soil_ is soil dust inhalation rate; EF is exposure frequency; ED is exposure duration; ABW is average body weight; PEF is soil dust particulate emission factor; ESSA is exposed skin surface area; SAF is soil adherence factor to skin; DAF is dermal absorption factor; T_avg_ is average exposure time. Relevant parameters for human health risk calculations are summarized in Table 2.

The carcinogenic risk index (CR, total carcinogenic risk TCR) and hazard index (HI) were calculated using Equations (9) and (10):

(9) $$\mathrm{HI}=\sum HQ_{i}=\sum \frac{{\mathrm{ADD}}_{i}}{{\mathrm{RfD}}_{i}}$$

(10) $$\mathrm{TCR}=\sum CR_{i}=\sum {\mathrm{ADD}}_{i}\times SF_{i}$$

where ADDi is average daily exposure dose of element i; RfDi is reference dose of PTE i for each exposure pathway; SFi is slope factor of PTE i for each exposure pathway. Risk grading criteria: no non-carcinogenic risk exists when HI < 1; adverse non-carcinogenic risks occur if HI ≥ 1. For carcinogenic risk: negligible risk at TCR < 10^−6^; low carcinogenic risk at 10^−6^ ≤ TCR < 10^−4^; high carcinogenic risk at TCR > 10^−4^. Parameters for each PTE are listed in Table 3.

Monte Carlo simulation has been widely adopted to quantify uncertainty in health risk results [46,47,48]. As a numerical simulation technique based on random sampling and probability statistics, it approximates solutions to complex problems through statistical analysis of large number of randomly generated samples. Consequently, Monte Carlo simulation has been extensively applied in numerical integration and stochastic process simulation. In this study, probabilistic distributions were assigned to the model input parameters (Table 4), and 10,000 iterative simulations were performed to generate random parameter values. The resulting outputs were used to estimate the probability distributions of the non-carcinogenic hazard index (HI) and total carcinogenic risk (TCR).

## 3. Results and Discussion

### 3.1. Soil Properties and Distribution Characteristics of PTEs in Soil

According to our recent research performed in the same region [50], the soil particle size composition around the uranium mine varied moderately across all sampling sites. The latest data (Supplemental Information, Figure S1 and Table S1) showed that silt dominated the soil particle composition, with a wide fraction of 56.0–91.2%, representing the predominant soil constituent. Sand and clay contents exhibited significantly lower proportions, ranging from 7.4 to 36.0% and 1.4–24.0%, respectively. These texture characteristics indicated that the soils were primarily silt-dominated soils, with minor variations in sand and clay proportions among different sampling locations. According to IUSS Working Group WRB. 2022 [51], most of the soil samples belonged to Spolic Technosol (Dystric, Siltic) and Dystric Regosol (Siltic). The total organic carbon (TOC) content showed substantial spatial heterogeneity, ranging from 0.49% to 6.90%. Most samples had moderate TOC levels. The total nitrogen (TN) content varied from 0.02% to 0.69%, showing a consistent variation trend with TOC content. The soil C/N ratio ranged from 7.34 to 24.55, with most values concentrated between 8 and 14, indicating relatively stable organic matter mineralization rates in most regional soils. The soil pH values ranged from 4.10 to 6.70, showing an overall acidic to weakly acidic environment. No neutral or alkaline soil was observed in the sampling area. Most soil samples presented strong acidity (pH < 5.5), indicating that mining-related activities have led to widespread acidification of local surface soils.

**Figure 2:**
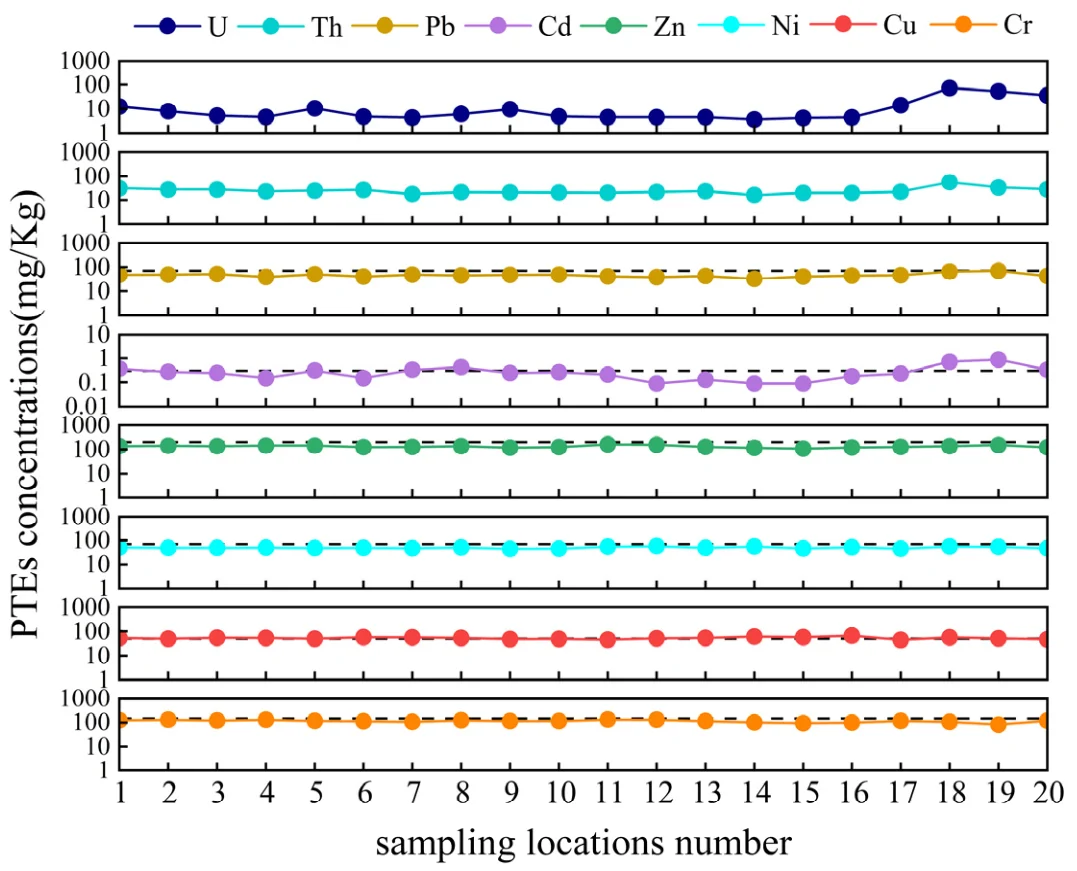
Statistical chart of PTEs concentrations in soil (the dotted line represents the risk screening value).

The statistical characteristics of PTE concentrations in the soils of the study area are shown in Figure 2. Except for Cu, U and Th, the concentrations of all other PTEs were below the risk screening values for agricultural soils specified in the National Soil Environmental Quality Standard (GB 15618-2018 [52], Table 5). The concentration ranges (average values) of the analyzed PTEs were as follows: Cr: 82.76–135.27 mg/kg (mean 115.79 mg/kg); Ni: 44.44–57.16 mg/kg (mean 49.85 mg/kg); Cu: 44.87–67.08 mg/kg (mean 53.05 mg/kg); Zn: 106.68–160.66 mg/kg (mean 133.14 mg/kg); Cd: 0.09–0.88 mg/kg (mean 0.29 mg/kg); Pb: 32.63–70.14 mg/kg (mean 46.61 mg/kg); Th: 16.21–55.74 mg/kg (mean 25.08 mg/kg); U: 3.64–73.61 mg/kg (mean 13.70 mg/kg). Since no official soil risk screening values were available for U and Th, only the remaining six elements were compared with the national standards. The mean concentrations of Cr, Ni, Cu, Zn, Cd, and Pb corresponded to 0.77, 0.71, 1.06, 0.66, 0.96 and 0.66 times their respective agricultural soil risk screening values, respectively.

The average concentrations of all eight PTEs exceeded the corresponding local soil background values by factors of 2.52 (Cr), 2.64 (Ni), 2.61 (Cu), 1.92 (Zn), 2.68 (Cd), 1.44 (Pb), 1.21 (Th) and 3.11 (U). The coefficient of variation (CV) was used to evaluate the spatial variability of PTE concentrations and indirectly reflect the influence of anthropogenic activities. The CV values followed the ordered: U (137.44%) > Cd (70.57%) > Th (33.91%) > Pb (18.43%) > Cr (11.74%) > Zn (10.66%) > Cu (10.21%) > Ni (7.39%). According to the CV classification, U and Cd exhibited high spatial variability (CV > 35%); Th showed moderate spatial variability (15% ≤ CV < 35%); whereas, the remaining elements displayed low variability. These results demonstrated that distribution of U, Cd and Th were strongly influenced by anthropogenic activities, resulting in pronounced spatial heterogeneity and localized enrichment within the study area.

The spatial distributions and variability of PTE were jointly controlled by natural geological processes and long-term uranium mining activities [10,34,53]. The elevated background concentrations of Cr, Ni, Cu, Zn, Pb and Th were primarily associated with regional lithology. Specifically, the Proterozoic metamorphic basement and Cretaceous volcanic cap rocks possess naturally elevated trace-element contents, leading to background concentrations that are generally higher that the local soil background values. In contrast, the extremely high coefficients of variation in U and Cd clearly indicate that their distribution were predominantly influenced by anthropogenic activities associated with uranium mining and tailings stockpiling [10,12]. Weathering of uranium-bearing ore and the continuous leaching of tailings impoundments released substantial amounts of U and Cd into surrounding soils through surface runoff and atmospheric dust deposition, resulting in localized hotspots with elevated concentrations, particularly near tailings dams and drainage channels [54].

Th exhibited moderate spatial variability, suggesting that its distribution was controlled by both natural geological sources and mining activities. Its natural occurrence in the parent bedrock established baseline concentration; whereas, the discharge of mining wastewater further elevated Th concentration in downslope agricultural soils. In contrast, elements with relatively low CV values (Cr, Ni, Cu, Zn, Pb) appeared to be less affected by mining activities, and their spatial distribution remained largely controlled by pedogenic and lithogenic processes. Their concentrations also remained below national risk screening values for agricultural soils. Overall, regional lithology and soil-forming processes established the natural geochemical baseline of PTEs; whereas, uranium mining represented the dominant anthropogenic driver that enhanced the spatial heterogeneity of toxic elements (esp. U and Cd) and promoted the formation of localized soil contamination hotspots.

**Table 5:** Statistical characteristics of PTEs concentration in soil [55].

	Cr	Ni	Cu	Zn	Cd	Pb	Th	U
Min.	82.76	44.44	44.87	106.68	0.09	32.63	16.21	3.64
Max.	135.27	57.16	67.08	160.66	0.88	70.14	55.74	73.61
Mean	115.79	49.85	53.05	133.14	0.29	46.61	25.08	13.70
STD	13.59	3.69	5.41	14.19	0.20	8.59	8.51	18.83
CV (%)	11.74	7.39	10.21	10.66	70.57	18.43	33.91	137.44
Skew. coefficient	−0.71	0.48	0.70	0.24	1.88	1.38	2.66	2.46
Kurt.	0.41	−0.65	1.00	−0.47	3.81	2.72	8.93	5.50
Background	45.9	18.9	20.3	69.4	0.108	32.3	20.7	4.4
Risk screening value	150	70	50	200	0.3	70	-	-

### 3.2. Source Identification of PTEs in Soil

#### 3.2.1. Correlation Analysis

**Figure 3:**
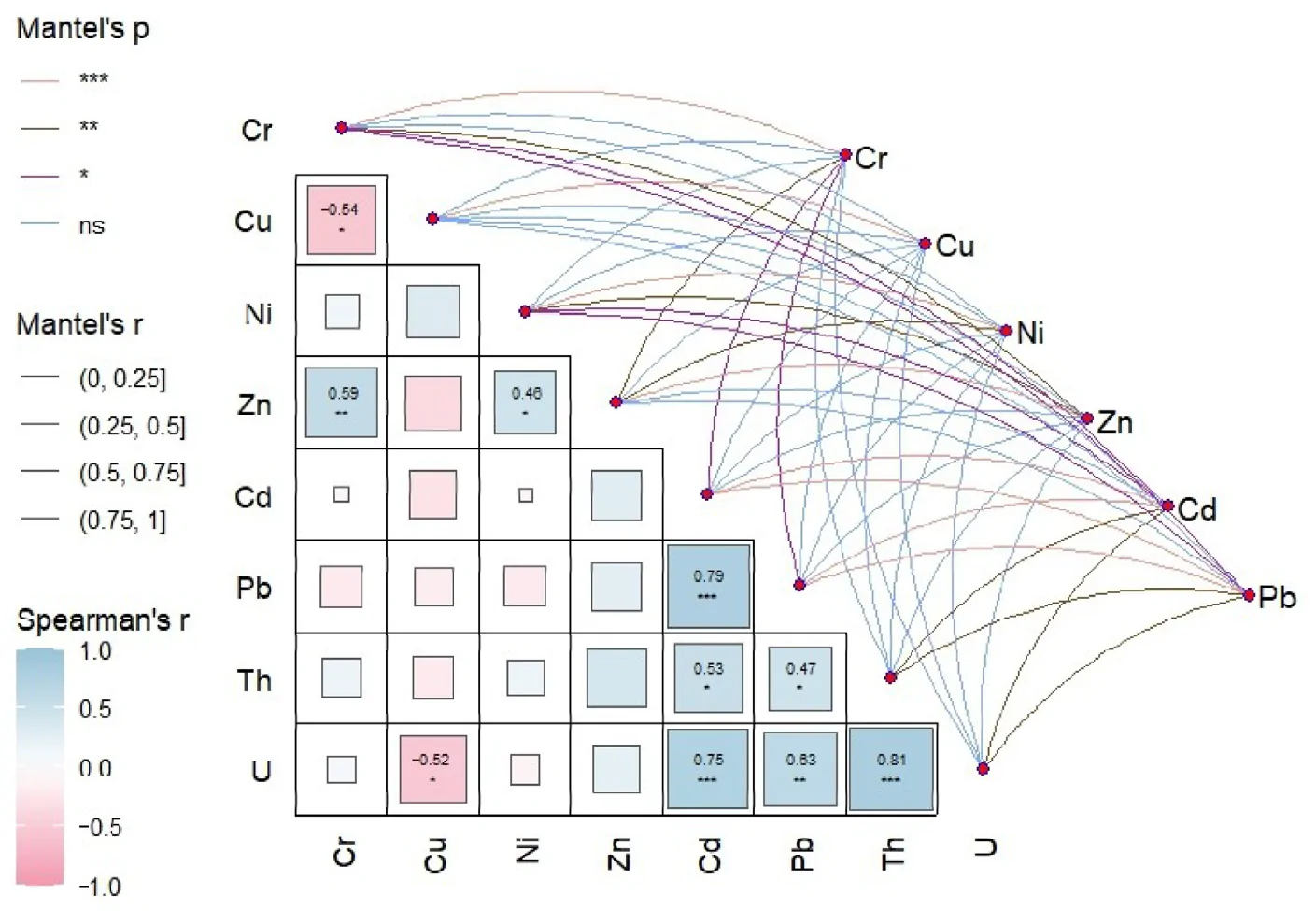
Correlation graph of potentially toxic elements in soil (*: *p* < 0.05; **: *p* < 0.01; ***: *p* < 0.001; ns: not significant).

Correlation analysis was conducted to examine the interrelationships among PTEs in the soil and to provide a basis for subsequent source apportionment. Significant correlations between different PTEs generally indicate that they originate from common pollution sources [47,56]. Pearson and Spearman are the two most widely used for evaluating relationships among environmental variables [57,58]. Because the distributions of U, Th, Cd, Pb and other elements did not satisfy the assumptions required for Pearson correlation analysis, Spearman correlation analysis was employed to investigate the relationships among PTEs in soil samples collected from the study area. As shown in the correlation heatmap (Figure 3), significant positive correlations were observed among Cd, Pb, U and Th, as well as between Zn and Cr (r > 0.5). In contrast, Zn exhibited only a weak positive correlation with Ni (r < 0.5), suggesting that these two elements may originate from multiple pollution sources. Therefore, further source apportionment analysis was required to discriminate their respective pollution sources contributions.

#### 3.2.2. Principal Component Analysis

The standardized dataset of soil PTEs was analyzed using SPSS 27. Prior to principal component analysis (PCA), the suitability of dataset was evaluated using the KMO measure and Bartlett’s test of sphericity. The KMO value was 0.523 (>0.5), and Bartlett’s test of sphericity was highly significant (*p* < 0.01). Eight variables (8 PTEs) and a sample size of 37 (*n* = 37) were employed for PCA. Two principal components with eigenvalues greater than 1 were extracted, explaining a cumulative 72.6% of the total variance (Table 6), which adequately represented the information contained in the original dataset.

The first principal component (PC1) accounted for 36.576% of the total variance, and exhibited strong positive loadings for Cd, Pb, Th and U. Combined with the correlation analysis, these four elements were inferred to originate primarily from a common pollution source. As shown in Table 5, the average concentrations of the four elements substantially exceeded the corresponding soil background values and were associated with relatively high coefficients of variation, implying strong anthropogenic influence. The agricultural land is located downstream of the tailings impoundment, making it susceptible to contamination from mining activities. During uranium ore mining, stockpiling and transportation, PTEs including Cd, Th and U can be transported to adjacent farmlands via rainwater erosion and surface runoff, resulting in accumulation in agricultural soils. This interpretation is consistent with the findings of Dai et al. [59]. In addition, previous studies have shown that Pb can also be released into surrounding soils during mining and ore-processing activities [60]. Accordingly, considering the mining history and elemental spatial characteristics, PC1 was identified as representing the mining and metallurgical activity source.

The second principal component (PC2) contributed 36.039% of the total variance, and showed strong positive loadings for Cr, Ni and Zn. As presented in Table 5, the mean concentrations of these three PTEs were markedly higher than the corresponding local soil background values. Previous studies have demonstrated that Cr was predominantly controlled by natural factors such as soil environmental evolution and parent materials [61,62,63]. Nevertheless, the elevated average Cr concentration observed in this study suggests additional anthropogenic inputs, including long-term application of Cr-containing pesticides and accumulation of Cr-bearing plastic waste associated with agricultural activities. Zn also exhibited a relatively high spatial variability, indicating the influence of human activities. Zn-bearing minerals may be released into surrounding soils, consistent with the findings of Han et al. [64]. Therefore, PC2 was interpreted as a mixed anthropogenic and natural source, potentially influenced by agricultural activities and geological processes.

#### 3.2.3. APCS-MLR Model Analysis

Based on PCA results, the factor scores were transformed into absolute principal component scores (APCS). Multiple linear regression models were then established using the APCS values as independent variables and measured concentration s of soil PTEs as dependent variables to quantify the contributions of different pollution sources. The model performance was evaluated by comparing the predicted and measured concentrations of each PTE, and coefficients of determination (R^2^) were calculated. The R^2^ values ranged from 0.637 to 0.847 for all PTEs, while the ratios of predicted to measured concentrations were close to 1, indicating good model performance and satisfactory predictive capability [37,65].

**Figure 4:**
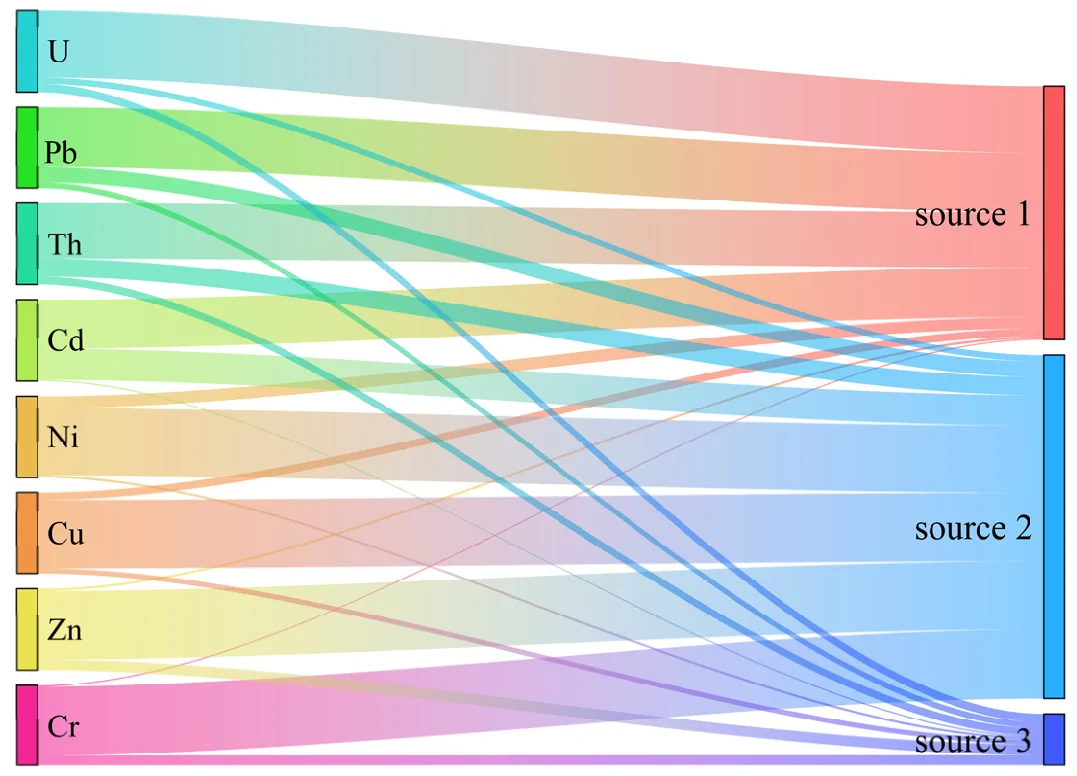
Contribution rate of PTEs pollution sources in soil.

The analytical results of the contribution ratios of different pollution sources to various PETs obtained from the APCS-MLR model are shown in Figure 4, Source 1 contributed predominantly to U, Pb and Th, and was therefore identified as the mining-metallurgical source. Source 2 showed the highest contributions to Ni, Cu, Zn and Cr, and was classified as the mixed source associated with mining activities, natural geogenic processes, and agricultural practices. Source 3 represented the natural geogenic source, with Cr exhibiting the largest contribution from source 3. The dominant contribution of source 3 to Cr is consistent with previous evidence that Cr concentrations are largely controlled by parent materials and pedogenic processes. Therefore, source 3 was preliminarily identified as the natural source.

### 3.3. Source Apportionment-Based Ecological Risk Assessment

Because no official soil risk screening values were available for U and Th; thus, only Cr, Ni, Cu, Zn, Cd and Pb were included in the ecological risk assessment in this study. The mean single potential ecological risk coefficients ($E_{r}^{i}$) for Cr, Ni, Cu, Zn, Cd and Pb were 5.05, 13.19, 13.07, 1.92, 79.30 and 7.22, respectively, as calculated using the potential ecological risk index. Among these elements, Cr, Ni, Cu and Zn presented low single-factor ecological risks; whereas, Cd exhibited a moderate single-factor ecological risk. The comprehensive potential ecological risk index (RI) of soil samples ranged from 64.50 to 288.81 with an average value of 119.37. Approximately 85% of the soil samples were classified as having low ecological risk while the remaining 15% fell into the moderate risk category, indicating that the overall potential ecological risk in the study area was generally low.

**Figure 5:**
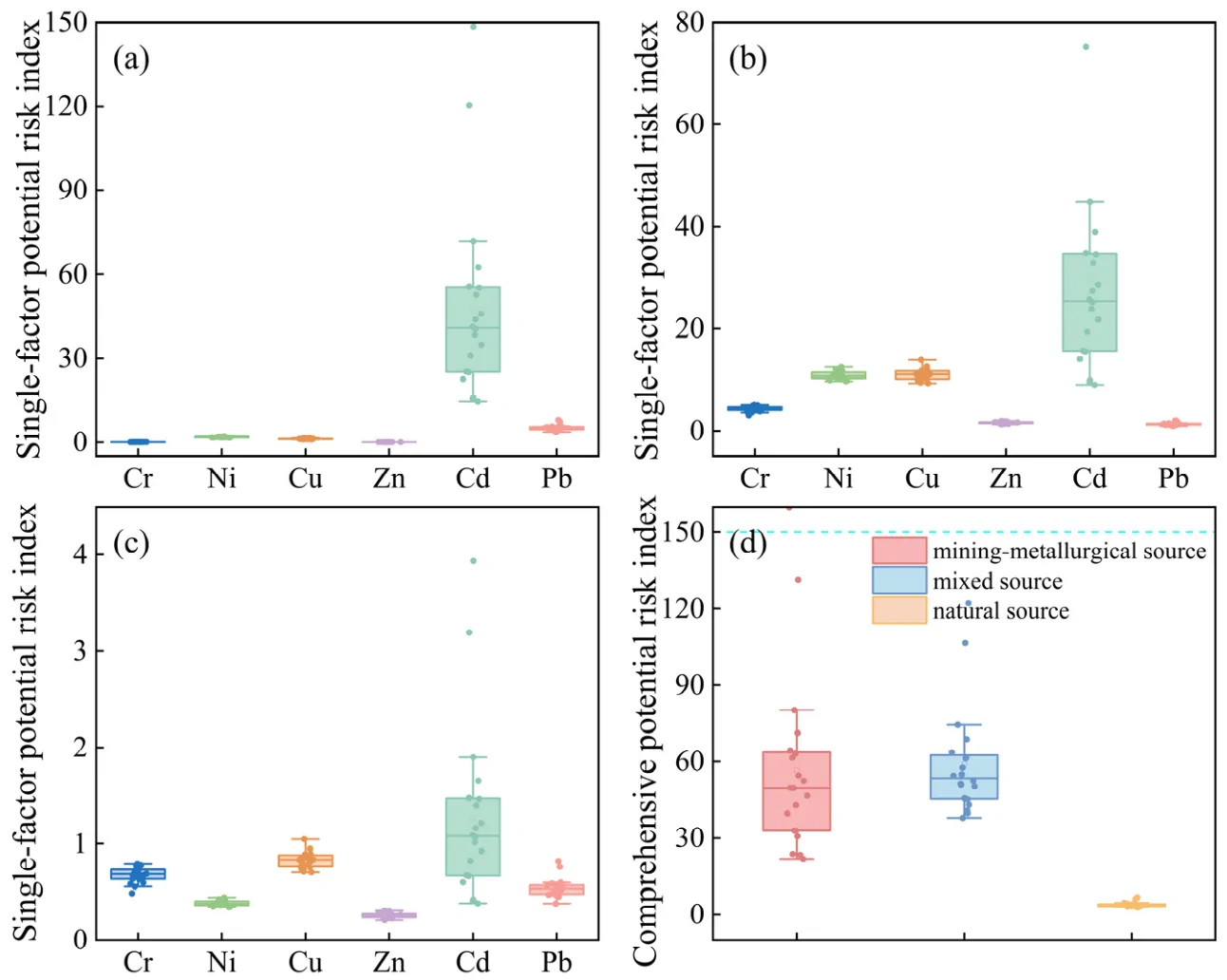
(**a**): Single-factor potential ecological index map of mining and metallurgical activity sources; (**b**): mixed-source single-factor potential ecological index map; (**c**): natural source single-factor potential ecological index map; (**d**): comprehensive potential ecological risk index chart from different sources (The dot line represents the low to moderate risk boundary).

The source-specific single ecological risks of PTEs are presented in Figure 5a–c. The results revealed that the average single-factor ecological risk index of Cd contributed by mining-metallurgical sources exceeded 40, corresponding to moderate risk. In contrast, the mean Cd risk coefficients associated with the other two sources were below 40 and were therefore classified as low ecological risk. In terms of integrated ecological risk index (RI) for the three pollution sources is presented in Figure 5d; where the dashed lines in each subplot indicate the threshold between low and moderate risk. The ecological risk contributions of three sources followed the order: mixed source (57.6) > mining-metallurgical source (48.3) > natural source (5.8). The mixed source contributed the largest proportion of overall ecological risks, with Cd identified as the primary risk factor. This pattern can be explained by two key mechanisms [23,66]. Firstly, Cd was one of the major PTEs released from mining and metallurgical activities as well as agricultural practices. Secondly, Cd has a toxic response factor of 30, which is 3–15 times higher than those of other target elements. Consequently, Cd contributed disproportionately to the total ecological risk, even when its concentration was relatively low. Furthermore, soil physicochemical properties (e.g., pH, organic matter content) and the chemical speciation of PTEs may further influence their ecological hazards. These factors should therefore be incorporated into future ecological risk assessments. Collectively, the results indicate that effective control of anthropogenic Cd emissions should be prioritized to reduce ecological risks in the soils of the uranium mining area.

### 3.4. Source Apportionment-Based Health Risk Assessment

**Figure 6:**
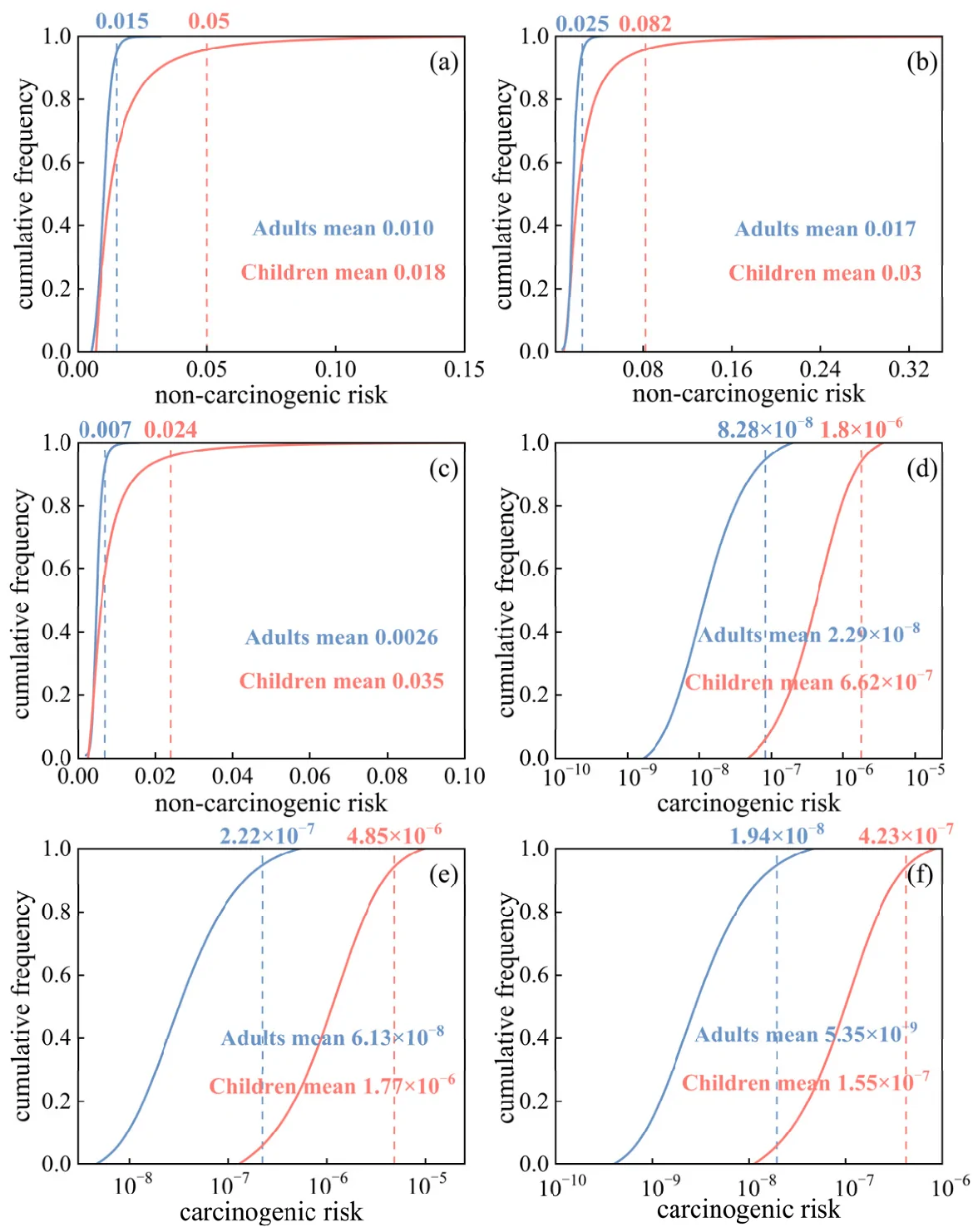
Probability distribution of non-carcinogenic and carcinogenic risks of PTEs from different sources ((**a**): non-carcinogenic risk of mining and metallurgy activities; (**b**): non-carcinogenic risk of mixed sources; (**c**): natural non-carcinogenic risk; (**d**): carcinogenic risk of mining and metallurgy activities; (**e**): carcinogenic risk of mixed sources; (**f**): natural cancer risk. Given that only total Cr concentration was determined in this study, the estimated Cr carcinogenic values are upper-bound values and do not represent the actual risk of Cr(VI)).

The probability distribution of health risks of PTEs in soils from different sources is shown in Figure 6. The dashed line in the figures represented the 95% comprehensive non-carcinogenic/carcinogenic risk hazard index values for children and adults from different sources. The Monte Carlo simulation using a “crystal ball” to evaluate the non-carcinogenic hazard quotients (Table 7) and carcinogenic risks (Table 8) posed by soil PTEs from distinct pollution sources for adults and children via three exposure pathways (oral ingestion, inhalation, and dermal contact). The results demonstrated that children consistently exhibited higher non-carcinogenic hazard quotients and carcinogenic risks across all exposure pathways compared with adults. This disparity is mainly attributed to children’s higher exposure intake relative to body weight, incomplete physiological development, and relatively weaker tolerance to environmental pollutants [67,68].

Although the average non-carcinogenic hazard quotients of PTEs from all pollution sources were below the safety threshold of 1.0, indicating acceptable non-carcinogenic risks, several health concerns remain. Specifically, Pb and Cd originating from mining and metallurgical sources induced carcinogenic risks within the tolerable range for both adults and children.

Human health risks were closely associated with soil PTEs concentrations. Such as, the maximum Cr concentration in the investigated region reached 135.27 mg/kg. Spatial analysis further indicated that areas in close proximity to tailings impoundments exhibited the highest Cr concentrations. These elevated risks were jointly controlled by the natural release of Cr through weathering of parent materials and additional anthropogenic inputs associated with uranium mining activities [69,70].

It also should be pointed out that the source specific health risk assessment used in this study was a quantitative method provided for identifying priority pollution sources, and there was a certain degree of uncertainty. Because the differences in pollutant bioavailability, potential interactions between multiple elements, and source-dependent exposure characteristics were not taken into account. Future research needs to integrate bioavailability measurements and more detailed toxicological mechanisms to further improve risk assessment of specific sources.

### 3.5. Identification of Priority Control Pollutants

**Figure 7:**
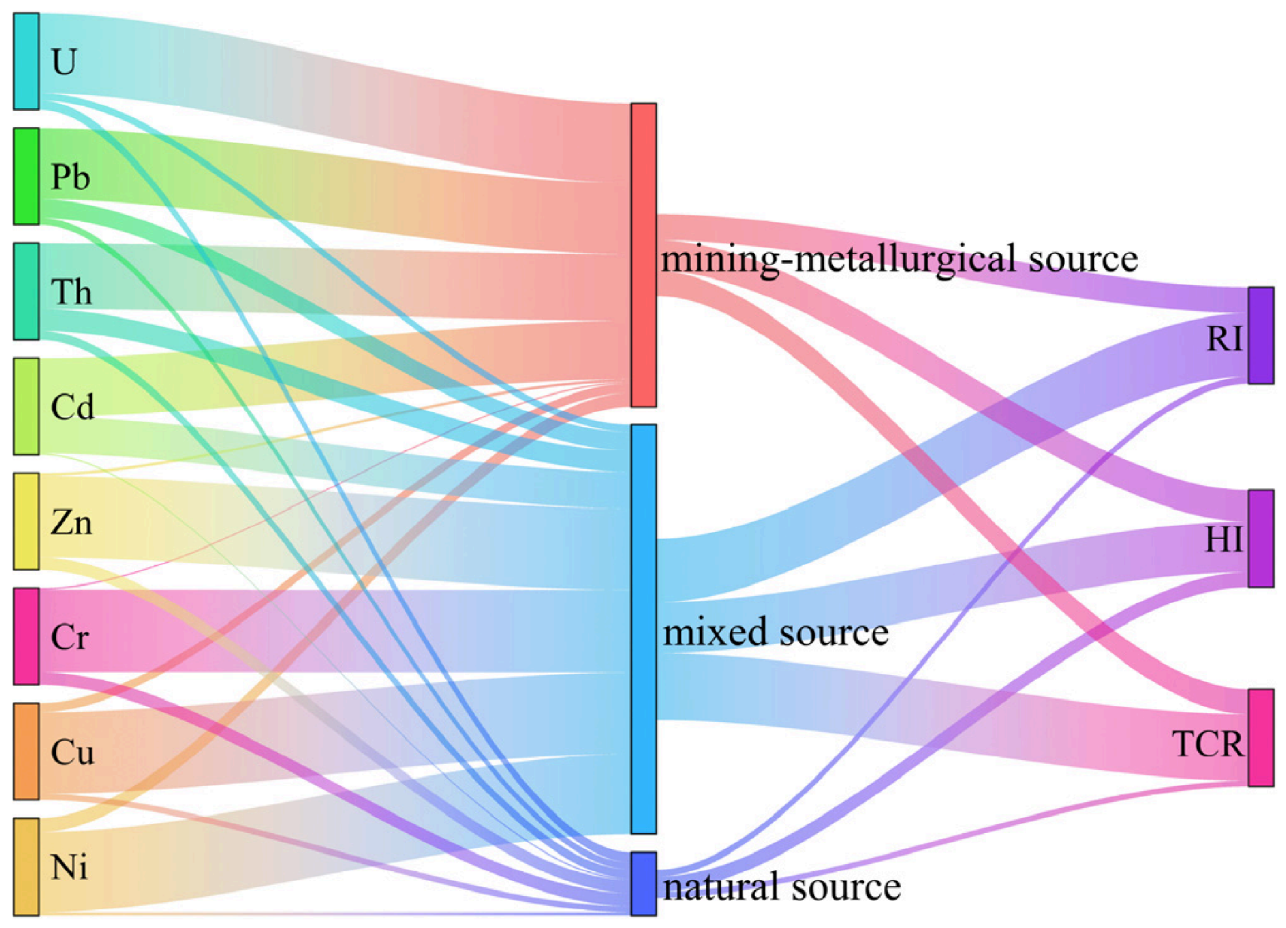
Relationship between sources and risks of PTEs in soil.

The linkage among pollution sources of PTEs, ecological risks and human health risks in the study area is exemplified in Figure 7. From the perspective of ecological risk, the mining and metallurgical sources and the mixed sources were dominant contributors, jointly accounting for 88.7% of total ecological risk, with Cd identified as the primary ecological pollutant. In terms of health risks, the mixed sources contributed the largest proportions of both non-carcinogenic and carcinogenic risks, accounting for 52.56% and 67.5% respectively.

Overall, based on the above statistical associations, Cd originating from the mining and metallurgical sources was associated with high ecological risks but relatively low health risks. In contrast, Cr derived from mixed sources posed comparatively low ecological hazards. These findings are consistent with those reported by Ma et al. [38], who showed that natural sources contributed minimally to soil Cd contamination. Similarly, Lei et al. [71] identified Cd as the priority pollutant for ecological risk; whereas, Cr was the dominant contributor to health risks in mining areas of Southwestern China. According to data from the Jiangxi Provincial Bureau of Statistics, the province has used approximately 130,000 tons of phosphate fertilizer annually in the past four years. Moreover, studies have shown that phosphate fertilizers can introduce Cd into agricultural soils [72,73]. Therefore, the Cd enrichment in the soil of the study area was ultimately considered to be related to mining related activities, but additional contributions from agricultural activities cannot be ruled out. These results demonstrated that targeted risk management strategies should be developed by comprehensively considering the source apportionment, pollution concentration, and toxicity differences in PTE. On-site emission data and elemental species should also be considered. In contrast, Cd contributed relatively little to the overall human health risk because of its comparatively low reference dose (RfD) and carcinogenic slope factor (SFi) adopted in the health risk assessment model [74].

Based on these findings, Cr derived from the mixed sources should be regarded as the top-priority pollutant for pollution control, and targeted measures should be implemented to reduce its associated contamination. It must be noted that, since actual Cr speciation are not obtained, this management recommendation was solely derived from upper-bound estimates based on total Cr measurements, which may lack a scientific basis. However, the long-term ecological impacts of Cd originating from mining-metallurgical sources should be continuously monitored to prevent its accumulation and biomagnification through the soil-food chain.

### 3.6. Limitations

Although this study quantified source contributions and coupled ecological and human health risks of PTEs in soils around a uranium mining area using APCS-MLR, potential ecological risk index and Monte Carlo simulation, several inherent limitations exist. Firstly, the risk evaluation only focused on chemical toxicity of PTEs rather than radiological hazards. Thus, we determined U and Th concentrations in soils, but did not carry out quantitative radiological risk assessment linked to radionuclides such as ^238^U, ^226^Ra and ^232^Th. Future work should collect radioactivity activity data and conduct integrated chemical and radiological risk evaluation. Secondly, only total Cr concentrations were determined via ICP-MS, while speciation analysis was not done to distinguish Cr(III) from Cr(VI). Given that carcinogenic parameters adopted in health risk calculation were specific to Cr(VI), the estimated carcinogenic risks associated with total Cr carried uncertainties. Thus, the interpretations regarding Cr health risks should be interpreted prudently, and they do not represent the actual Cr(VI) risk. Subsequent sampling campaigns will include sequential extraction or speciation testing to differentiate Cr species. Moreover, as direct Cr speciation data are unavailable in the present work, the aforementioned management recommendations rely exclusively on upper-bound estimates calculated from total Cr concentrations. Future investigations focusing on Cr speciation analysis are required to establish a robust scientific foundation for such suggestions. Thirdly, the KMO value (0.523) for PCA was a bit lower than the commonly recommended threshold of 0.6. Two principal components were initially extracted according to standard criteria; whereas, a third component was retained in the APCS-MLR modeling workflow. This might introduce uncertainty into source identification results. PCA/APCS-MLR is a statistical receptor model that provides speculative source discrimination rather than definitive source proof. Reliable source verification requires auxiliary evidence including mineralogical characterization, pollutant species, field emission monitoring and geochemical background surveys. Finally, the management suggestions proposed in this study were derived from statistical source apportionment and chemical risk calculation. Due to the above uncertainties related to PCA performance, undifferentiated Cr species, and absence of radiological risk assessment, relevant policy recommendations should be regarded as preliminary references. Future research that combines speciation analysis, isotope tracing, and field emission monitoring should be done to further validate the present findings.

## 4. Conclusions

This work established an integrated methodological framework that linked source apportionment to probabilistic risk assessment, by combining APCS-MLR receptor model and Monte Carlo-based probabilistic ecological and human health risk assessment. The proposed framework was successfully applied to source apportionment and risk assessment for PTEs-contaminated soils in a uranium mining area of southern Jiangxi province. Based on statistical inference, three potential pollution sources of soil PTEs were identified: a mining and metallurgical source, a mixed source composed of mining activities and agricultural activities, and a naturally occurring source. Source-specific risk quantification preliminarily suggested distinct ecological and human health risk profiles among the identified pollution sources. Cd, which originated primarily from mining and metallurgical activities, was the dominant contributor to regional ecological risks because of its extremely high toxic response factor. However, under the exposure scenarios considered in this study, Cd posed relatively low human health risks. It must be noted that, although Cr was identified as a health-risk pollutant based on its total concentration measurements, these outcomes do not represent the actual risk from Cr(VI), since Cr speciation analyses were not performed in the present study. Notwithstanding, our findings demonstrate that effective soil pollution management in uranium mining areas requires an integrated framework that combines source apportionment, spatial distribution of contamination, species-specific toxic properties and differentiated hazard endpoints encompassing both ecological and human health risks. Such an approach enables the identification of priority pollution based not only on their source specific risk contributions. Furthermore, site-specific pollutants (e.g., Cd) should be prioritized to support long-term soil risk management and targeted pollution control in uranium mining watersheds.

## Figures and Tables

**Table 1:** The evaluation criteria of the potential ecological risk index (RI).

Potential Ecological Risk Index (E_i_)	Potential Ecological Risk Index (RI)	Ecological Hazard Level
Ei < 40	RI < 150	low
40 ≤ E_i_ < 80	150 ≤ RI < 300	moderate
80 ≤ E_i_ < 160	300 ≤ RI < 600	high
160 ≤ E_i_	600 ≤ RI	extremely high

**Table 2:** Parameters used in the human health risk model.

Parameters	Meaning	Unit	Adults	Children
IngR	ingestion rate	mg/d	100	200
InhR_soil_	inhalation rate	m^3^/d	12.8	7.63
EF	Exposure frequency	d/year	350	
ED	Exposure duration	year	30	6
ABW	Average body weight	kg	70	15
PEF	Particulate emission factor	m^3^/kg	1.36 × 10^9^	
ESSA	Exposed skin surface area	cm^2^	5700	2800
SAF	Soil adherence factor	cm^−2^∙h^−1^	0.07	0.2
DAF	Dermal absorption factor	-	0.001	
T_avg_	Average exposure time	d	Non-carcinogenic: ED × 365 Carcinogenic: 70 × 365	

Note: “-” indicates no data available. The same below.

**Table 3:** Reference dose (RfD) and slope factor (SF) of PTE in different exposure pathways.

PTEs	RfD (mg∙kg^−1^∙d^−1^)			SF_i_ (kg∙d∙mg^−1^)		
	Oral Ingestion	Inhalation	Dermal Contact	Oral Ingestion	Inhalation	Dermal Contact
Cr	3.00 × 10^−3^	2.86 × 10^−5^	6.00 × 10^−5^	0.501	42	20.0
Cu	4.00 × 10^−2^	4.02 × 10^−2^	1.20 × 10^−2^	-	-	-
Ni	2.00 × 10^−2^	2.06 × 10^−2^	5.40 × 10^−3^	-	0.84	
Zn	3.00 × 10^−1^	3.00 × 10^−1^	6.00 × 10^−2^	-	-	-
Cd	1.00 × 10^−3^	1.00 × 10^−5^	1.00 × 10^−5^		6.3	
Pb	3.50 × 10^−3^	3.52 × 10^−3^	5.25 × 10^−4^	-	-	-

**Table 4:** Exposure dose simulation parameters [49].

Parameters	Distribution Type	Unit	Adults	Children
IngR	lognormal distribution	mg/d	(16.57, 4.05)	(7.19, 1.62)
InhR_soil_	triangular distribution	m^3^/d	(4, 30, 52)	(66, 103, 161)
EF	triangular distribution	d/year	(180, 345, 365)	
ABW	uniform distribution (adults) lognormal distribution (children)	kg	(55.7, 68.6)	(37.0, 2.98)
ESSA	triangular distribution	cm^2^	(760, 1530, 3820)	(430, 860, 2160)
SAF	lognormal distribution	cm^−2^∙h^−1^	(0.65, 1.20)	(0.49, 0.54)

**Table 6:** Potentially toxic elements load factor and variance contribution rates.

PTEs	Components	
	PC1	PC2
Cr	0.042	0.972
Cu	−0.392	0.699
Ni	−0.318	0.763
Zn	−0.079	0.957
Cd	0.732	0.197
Pb	0.911	0.103
Th	0.713	0.181
U	0.755	0.253
Contribution (%)	36.576	36.039

**Table 7:** Non-carcinogenic risk of PTEs from different sources.

PTEs	Mining Source		Mixed Source		Natural Source	
	Adults	Children	Adults	Children	Adults	Children
Cr	8.46 × 10^−3^	1.48 × 10^−2^	5.36 × 10^−2^	9.35 × 10^−2^	9.82 × 10^−4^	1.71 × 10^−3^
Cu	1.14 × 10^−4^	2.15 × 10^−4^	1.52 × 10^−3^	2.86 × 10^−3^	1.71 × 10^−4^	3.23 × 10^−4^
Ni	9.92 × 10^−5^	1.87 × 10^−4^	2.81 × 10^−3^	5.29 × 10^−3^	4.93 × 10^−4^	9.28 × 10^−4^
Zn	8.24 × 10^−5^	1.46 × 10^−4^	5.19 × 10^−4^	9.22 × 10^−4^	1.81 × 10^−5^	3.22 × 10^−5^
Cd	3.33 × 10^−4^	5.70 × 10^−4^	2.08 × 10^−4^	3.55 × 10^−4^	8.83 × 10^−6^	1.51 × 10^−5^
Pb	1.40 × 10^−2^	2.50 × 10^−2^	3.60 × 10^−3^	6.45 × 10^−3^	1.43 × 10^−3^	2.56 × 10^−3^

**Table 8:** Carcinogenic risks of PTEs from different sources.

PTEs	Mining Source		Mixed Source		Natural Source	
	Adults	Children	Adults	Children	Adults	Children
Cr	4.99 × 10^−10^	1.11 × 10^−7^	2.72 × 10^−8^	6.04 × 10^−6^	4.30 × 10^−9^	9.53 × 10^−7^
Ni	4.93 × 10^−11^	1.88 × 10^−8^	2.81 × 10^−10^	1.07 × 10^−7^	9.92 × 10^−12^	3.79 × 10^−9^
Cd	3.37 × 10^−11^	1.36 × 10^−10^	7.94 × 10^−10^	3.21 × 10^−9^	1.27 × 10^−9^	5.15 × 10^−9^

## Data Availability

The original contributions presented in this study are included in the article/Supplementary Material. Further inquiries can be directed to the corresponding authors.

## Supplementary Materials

The following supporting information can be downloaded at: https://www.mdpi.com/article/10.3390/toxics14090748/s1, Figure S1: Distribution of sampling sites in study area; Table S1: Physicochemical soil parameters.

## References

[1] Hou D., Jia X., Wang L., McGrath S.P., Zhu Y.-G., Hu Q., Zhao F.-J., Bank M.S., O’Connor D., Nriagu J. (2025). Global soil pollution by toxic metals threatens agriculture and human health. Science.

[2] Siddig M.M.S., Brevik E.C., Sauer D. (2025). Human health risk assessment from potentially toxic elements in the soils of Sudan: A meta-analysis. Sci. Total Environ..

[3] Ondrasek G., Shepherd J., Rathod S., Dharavath R., Rashid M., Brtnicky M., Shahid M.S., Horvatinec J., Rengel Z. (2025). Metal contamination—A global environmental issue: Sources, implications & advances in mitigation. RSC Adv..

[4] Adnan M., Xiao B., Xiao P., Zhao P., Li R., Bibi S. (2022). Research Progress on Heavy Metals Pollution in the Soil of Smelting Sites in China. Toxics.

[5] Danes J.M., Palma F.R., Bonini M.G. (2021). Arsenic and other metals as phenotype driving electrophiles in carcinogenesis. Semin. Cancer Biol..

[6] Bembamba M., Sako A. (2025). Contamination status and toxicity risk assessment of selected potentially toxic elements in surface soils under the influence of different land uses in Midwestern Burkina Faso, West Africa. J. Trace Elem. Miner..

[7] Galhardi J.A., de Mello J.W.V., Wilkinson K.J. (2020). Environmental and health risk assessment of agricultural areas adjacent to uranium ore fields in Brazil. Environ. Geochem. Health.

[8] Li H., Wang Q., Zhang C., Su W., Ma Y., Zhong Q., Xiao E., Xia F., Zheng G., Xiao T. (2024). Geochemical Distribution and Environmental Risks of Radionuclides in Soils and Sediments Runoff of a Uranium Mining Area in South China. Toxics.

[9] Haghighizadeh A., Rajabi O., Nezarat A., Hajyani Z., Haghmohammadi M., Hedayatikhah S., Asl S.D., Beni A.A. (2024). Comprehensive analysis of heavy metal soil contamination in mining Environments: Impacts, monitoring Techniques, and remediation strategies. Arab. J. Chem..

[10] Zhang Z., Tang Z., Liu Y., He H., Guo Z., Feng P., Chen L., Sui Q. (2023). Study on the Ecotoxic Effects of Uranium and Heavy Metal Elements in Soils of a Uranium Mining Area in Northern Guangdong. Toxics.

[11] Anamika K., Mehra R., Malik P. (2020). Assessment of radiological impacts of natural radionuclides and radon exhalation rate measured in the soil samples of Himalayan foothills of Uttarakhand, India. J. Radioanal. Nucl. Chem..

[12] Chen L., Wang J., Beiyuan J., Guo X., Wu H., Fang L. (2022). Environmental and health risk assessment of potentially toxic trace elements in soils near uranium (U) mines: A global meta-analysis. Sci. Total Environ..

[13] Zhou Z., Yang Z., Sun Z., Liao Q., Guo Y., Chen J. (2020). Multidimensional pollution and potential ecological and health risk assessments of radionuclides and metals in the surface soils of a uranium mine in East China. J. Soils Sediments.

[14] Wei X., Xu N., Chen L., Chen J., Li J. (2024). Occurrence, mobility, and potential risk of uranium in an abandoned stone coal mine of Jiangxi Province, China. Environ. Earth Sci..

[15] Ouyang J., Liu Z., Zhang L., Wang Y., Zhou L. (2020). Analysis of influencing factors of heavy metals pollution in farmland-rice system around a uranium tailings dam. Process Saf. Environ. Prot..

[16] Xiang L., Liu P.-h., Jiang X.-f., Chen P.-j. (2019). Health risk assessment and spatial distribution characteristics of heavy metal pollution in rice samples from a surrounding hydrometallurgy plant area in No. 721 uranium mining, East China. J. Geochem. Explor..

[17] Jia Y., Zhang W., Liu M., Peng Y., Hao C. (2022). Spatial Distribution, Pollution Characteristics and Source of Heavy Metals in Farmland Soils around Antimony Mine Area, Hunan Province. Pol. J. Environ. Stud..

[18] Lu Z., Liu Z. (2018). Pollution characteristics and risk assessment of uranium and heavy metals of agricultural soil around the uranium tailing reservoir in Southern China. J. Radioanal. Nucl. Chem..

[19] Yang Z., Yu J., Yang K., Zhang Q., Chen Y., Qiao S. (2024). Source Apportionment and Risk Assessment of Potentially Toxic Elements Based on PCA and PMF Model in Black Soil Area of Hailun City, Northeast China. Toxics.

[20] Cui W., Mei Y., Liu S., Zhang X. (2023). Health risk assessment of heavy metal pollution and its sources in agricultural soils near Hongfeng Lake in the mining area of Guizhou Province, China. Front. Public Health.

[21] Cai A., Zhang H., Zhao Y., Wang X., Wang L., Zhao H. (2022). Quantitative source apportionment of heavy metals in atmospheric deposition of a typical heavily polluted city in Northern China: Comparison of PMF and UNMIX. Front. Environ. Sci..

[22] Cao L., Duan H., Cheng B., Xiang Q., Wang S., Fu Z., Xu X., Ren Q., Yang H., Yu Y. (2025). Sources and health risks of heavy metal(loid) contamination in farmland near Shanxi coal mines. Front. Soil Sci..

[23] Li W., Cao X., Hu Y., Cheng H. (2024). Source Apportionment and Risk Assessment of Heavy Metals in Agricultural Soils in a Typical Mining and Smelting Industrial Area. Sustainability.

[24] Zhou W., Yu R., Guo F., Shen C., Liu Y., Huang Y. (2024). Source apportionment and risk assessment of soil heavy metals in the Huangshui River Basin using a hybrid model. Ecol. Indic..

[25] Liu Z., Zeng Y., Wang W., Zhang B., Liu J., Zhao X. (2026). Coupling positive matrix factorization with machine learning to quantify contamination sources and map health risks of heavy metals in karst soils. Soil Environ. Health.

[26] Gan L., Wang J., Xie M., Yang B. (2022). Ecological risk and health risk analysis of soil potentially toxic elements from oil production plants in central China. Sci. Rep..

[27] Wu Y., Xia Y., Mu L., Liu W., Wang Q., Su T., Yang Q., Milinga A., Zhang Y. (2024). Health Risk Assessment of Heavy Metals in Agricultural Soils Based on Multi-Receptor Modeling Combined with Monte Carlo Simulation. Toxics.

[28] Chernysh Y., Chubur V., Ablieieva I., Skvortsova P., Yakhnenko O., Skydanenko M., Plyatsuk L., Roubík H. (2024). Soil Contamination by Heavy Metals and Radionuclides and Related Bioremediation Techniques: A Review. Soil Syst..

[29] Liu Z., Du Q., Guan Q., Luo H., Shan Y., Shao W. (2023). A Monte Carlo simulation-based health risk assessment of heavy metals in soils of an oasis agricultural region in northwest China. Sci. Total Environ..

[30] Huang J., Wu Y., Sun J., Li X., Geng X., Zhao M., Sun T., Fan Z. (2021). Health risk assessment of heavy metal(loid)s in park soils of the largest megacity in China by using Monte Carlo simulation coupled with Positive matrix factorization model. J. Hazard. Mater..

[31] Wang W., Chen C., Liu D., Wang M., Han Q., Zhang X., Feng X., Sun A., Mao P., Xiong Q. (2022). Health risk assessment of PM2.5 heavy metals in county units of northern China based on Monte Carlo simulation and APCS-MLR. Sci. Total Environ..

[32] Yu X., Su X., Wang Z., Hou Z., Li B., Deng T., Yan Z. (2025). Genesis of the Xiangshan Uranium Ore Field: Implications from Tescan Integrated Mineral Analyzer and Micro-X-Ray Fluorescence Mapping and Thermodynamic Modeling. Minerals.

[33] Wang Y., Fan H., Pang Y., Xiao W. (2022). Geochemical Characteristics of Chlorite in Xiangshan Uranium Ore Field, South China and Its Exploration Implication. Minerals.

[34] Guo F., Li Z., Deng T., Qu M., Zhou W., Huang Q., Shang P., Zhang C., Yan Z. (2020). Key factors controlling volcanic-related uranium mineralization in the Xiangshan Basin, Jiangxi Province, South China: A review. Ore Geol. Rev..

[35] Huang X., Hui Z. (2026). Analysis of Vegetation Ecological Anomaly Response in the Xiangshan Uranium Mining Area Based on Multi-Source Remote Sensing Data Fusion. Forests.

[36] (2016). Solid waste—Determination of 22 Metal Elements—Inductively Coupled Plasma Optical Emission Spectrometry.

[37] Gong C., Xia X., Lan M., Shi Y., Lu H., Wang S., Chen Y. (2024). Source identification and driving factor apportionment for soil potentially toxic elements via combining APCS-MLR, UNMIX, PMF and GDM. Sci. Rep..

[38] Ma J., Shen Z.-J., Wang S.-L., Deng L., Sun J., Liu P., She Z.-L. (2023). Source apportionment of heavy metals in soils around a coal gangue heap with the APCS-MLR and PMF receptor models in Chongqing, southwest China. J. Mt. Sci..

[39] Jianfei C., Chunfang L., Lixia Z., Quanyuan W., Jianshu L. (2020). Source apportionment of potentially toxic elements in soils using APCS/MLR, PMF and geostatistics in a typical industrial and mining city in Eastern China. PLoS ONE.

[40] Liu H., Ma J., Taj R., Xu M., Lou F., Liu W., Xu Y., Xu J., Xu Y., Liu D. (2024). Quantitative assessment of ecological risk from pollution source based on geostatistical analysis and APCS-MLR model. Environ. Sci. Pollut. Res..

[41] Zhao J., Cao C., Chen X., Zhang W., Ma T., Irfan M., Zheng L. (2024). Source-specific ecological risk analysis and critical source identification of heavy metal(loid)s in the soil of typical abandoned coal mining area. Sci. Total Environ..

[42] Zhang W.-H., Yan Y., Yu R.-L., Hu G.-R. (2021). The sources-specific health risk assessment combined with APCS/MLR model for heavy metals in tea garden soils from south Fujian Province, China. Catena.

[43] Chen D., Li X., Wang Z., Kang C., He T., Liu H., Jiang Z., Xi J., Zhang Y. (2024). Systematic assessment of source identification and ecological and probabilistic health risks of potentially toxic elements (PTEs) in soils of a typical coal mining area in Guanzhong region. Heliyon.

[44] Zhang S., Han Y., Peng J., Chen Y., Zhan L., Li J. (2023). Human health risk assessment for contaminated sites: A retrospective review. Environ. Int..

[45] Deliboran A., Varol M., Aytop H. (2024). Evaluation of ecological and health risks of trace elements in soils of olive orchards and apportionment of their sources using the APCS-MLR receptor model. Environ. Geochem. Health.

[46] Panqing Y., Abliz A., Xiaoli S., Aisaiduli H. (2023). Human health-risk assessment of heavy metal–contaminated soil based on Monte Carlo simulation. Sci. Rep..

[47] Ma J., Chen L., Chen H., Wu D., Ye Z., Zhang H., Liu D. (2023). Spatial distribution, sources, and risk assessment of potentially toxic elements in cultivated soils using isotopic tracing techniques and Monte Carlo simulation. Ecotoxicol. Environ. Saf..

[48] Jiang C., Zhao Q., Zheng L., Chen X., Li C., Ren M. (2021). Distribution, source and health risk assessment based on the Monte Carlo method of heavy metals in shallow groundwater in an area affected by mining activities, China. Ecotoxicol. Environ. Saf..

[49] U.S. Environmental Protection Agency (2011). Exposure Factors Handbook: 2011 Edition.

[50] Dong S. (2025). Enrichment Characteristics and Transport Behaviors of Rare Earth Elements, and Uranium and Thorium in Rhizosphere Soil-Plant Systems in Xiangshan Uranium Mine Tailings. Master’s Thesis.

[51] IUSS Working Group WRB, World Reference Base for Soil Resources (2022). International Soil Classification System for Naming Soils and Creating Legends for Soil Maps.

[52] (2018). Soil Environment Quality Risk Control Standard for Soil Contamination of Agriculture Land.

[53] Xia Q., Zhang L., Dong H., Li Z., Zhang Y., Hu J., Chen H., Chen Y. (2020). Bio-weathering of a uranium-bearing rhyolitic rock from Xiangshan uranium deposit, Southeast China. Geochim. Cosmochim. Acta.

[54] Ramadan R.S., Dawood Y.H., Yehia M.M., Gad A. (2022). Environmental and health impact of current uranium mining activities in southwestern Sinai, Egypt. Environ. Earth Sci..

[55] Xiao M., Xu S., Yang B., Zeng G., Qian L., Huang H., Ren S. (2022). Contamination, Source Apportionment, and Health Risk Assessment of Heavy Metals in Farmland Soils Surrounding a Typical Copper Tailings Pond. Int. J. Environ. Res. Public Health.

[56] Li Y., Wang S., Shang X., Zhou H., He J., Chen W., Wang L., Zhao X., Bao L., Zhang N. (2025). Characterization and source apportionment of heavy metal contamination in agricultural soils in the complex genesis region of western Yunnan. Sci. Rep..

[57] Schwarte L.A., Boer C., Schober P. (2018). Correlation Coefficients: Appropriate Use and Interpretation. Anesth. Analg..

[58] Sojka M., Jaskuła J. (2022). Heavy Metals in River Sediments: Contamination, Toxicity, and Source Identification—A Case Study from Poland. Int. J. Environ. Res. Public Health.

[59] Dai P.-F., Wang S., Qian K., Yuan F.-H., Zhu Y.-A., Huang D.-J. (2021). Analysis of Bioavailability of Heavy Metals in Farmland Soil in Uranium Mining Area. J. Ecol. Rural. Environ..

[60] Kříbek B., Nyambe I., Sracek O., Mihaljevič M., Knésl I. (2023). Impact of Mining and Ore Processing on Soil, Drainage and Vegetation in the Zambian Copperbelt Mining Districts: A Review. Minerals.

[61] Hu B., Shao S., Ni H., Fu Z., Hu L., Zhou Y., Min X., She S., Chen S., Huang M. (2020). Current status, spatial features, health risks, and potential driving factors of soil heavy metal pollution in China at province level. Environ. Pollut..

[62] Chai L., Wang Y., Wang X., Ma L., Cheng Z., Su L., Liu M. (2021). Quantitative source apportionment of heavy metals in cultivated soil and associated model uncertainty. Ecotoxicol. Environ. Saf..

[63] Shao S., Hu B., Tao Y., You Q., Huang M., Zhou L., Chen Q., Shi Z. (2022). Comprehensive source identification and apportionment analysis of five heavy metals in soils in Wenzhou City, China. Environ. Geochem. Health.

[64] Han W., Zhao R., Liu W., Wang Y., Zhang S., Zhao K., Nie J. (2023). Environmental contamination characteristics of heavy metals from abandoned lead–zinc mine tailings in China. Front. Earth Sci..

[65] Shi H., Zeng M., Peng H., Huang C., Sun H., Hou Q., Pi P. (2022). Health Risk Assessment of Heavy Metals in Groundwater of Hainan Island Using the Monte Carlo Simulation Coupled with the APCS/MLR Model. Int. J. Environ. Res. Public Health.

[66] Saleem M., Pierce D., Wang Y., Sens D.A., Somji S., Garrett S.H. (2024). Heavy Metal(oid)s Contamination and Potential Ecological Risk Assessment in Agricultural Soils. J. Xenobiotics.

[67] Bair E.C. (2022). A Narrative Review of Toxic Heavy Metal Content of Infant and Toddler Foods and Evaluation of United States Policy. Front. Nutr..

[68] Luo H., Wang P., Wang Q., Lyu X., Zhang E., Yang X., Han G., Zang L. (2024). Pollution sources and risk assessment of potentially toxic elements in soils of multiple land use types in the arid zone of Northwest China based on Monte Carlo simulation. Ecotoxicol. Environ. Saf..

[69] Yan T., Wang X., Liu D., Chi Q., Zhou J., Xu S., Zhang B., Nie L., Wang W. (2021). Continental-scale spatial distribution of chromium (Cr) in China and its relationship with ultramafic-mafic rocks and ophiolitic chromite deposit. Appl. Geochem..

[70] Banerjee S., Ghosh S., Prajapati J., Bhattacharyya P. (2025). Hidden threats beneath: Uncovering the bio-accessible hazards of chromite-asbestos mine waste and their impacts on rice components via multi-machine learning algorithm. Environ. Geochem. Health.

[71] Lei M., Li K., Guo G., Ju T. (2022). Source-specific health risks apportionment of soil potential toxicity elements combining multiple receptor models with Monte Carlo simulation. Sci. Total Environ..

[72] Liang H., Tan Y., Yin J., Peng Y., Wei M., Chen H., Chen Q. (2024). Phosphate Fertilizers’ Dual Role in Cadmium-Polluted Acidic Agricultural Soils: Dosage Dependency and Passivation Potential. Agronomy.

[73] Davidova S., Milushev V., Satchanska G. (2024). The Mechanisms of Cadmium Toxicity in Living Organisms. Toxics.

[74] Miletić A., Lučić M., Onjia A. (2023). Exposure Factors in Health Risk Assessment of Heavy Metal(loid)s in Soil and Sediment. Metals.

