# Supplementary material for: Source Apportionment-Based Assessment of Ecological and Health Risk for Potentially Toxic Elements in the Soil Around a Uranium Mine
Source: Toxics. 2026 Aug 25;14(9):748. doi: 10.3390/toxics14090748 (PMC13611104; doi:10.3390/toxics14090748)
Supplement: Supplementary file 1 [file toxics-14-00748-s001.zip › toxics-4486361-supplementary.pdf]

supplemental information

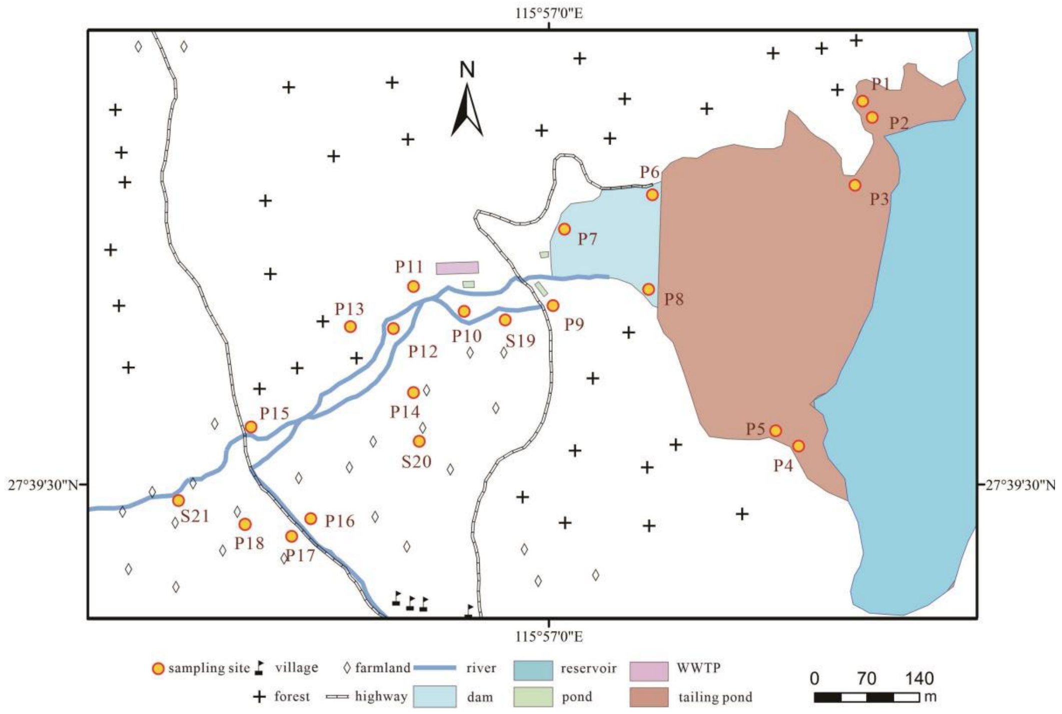

Figure S1. Distribution of sampling sites in study area.

Table S1. Physicochemical soil parameters.

Sample ID	Clay(%)	Silt(%)	Sand (%)	TOC (%)	TN (%)	C/N	pH	WRB 2022 soil name
P1	6	63.7	30.3	1.75	0.18	9.72	4.21	Spolic Technosol (Dystric, Siltic)
P2	24	66.2	9.8	1.48	0.16	9.25	4.10	Spolic Technosol (Dystric, Siltic)
P3	8	56	36	0.49	0.02	24.55	4.56	Spolic Technosol (Dystric, Siltic)
P4	18	63.6	18.4	0.71	0.05	14.10	4.78	Spolic Technosol (Dystric, Siltic)
P5	12	62.2	25.8	1.57	0.15	10.47	4.65	Spolic Technosol (Dystric, Siltic)
P6	10	62.7	27.3	0.90	0.07	12.81	5.37	Spolic Technosol (Eutric, Siltic)
P7	10	69.7	20.3	3.47	0.39	8.90	5.12	Spolic Technosol (Dystric, Siltic)
P8	13	72.7	14.3	1.77	0.15	11.80	6.70	Spolic Technosol (Eutric, Siltic)
P9	1.4	91.2	7.4	2.29	0.24	9.71	4.70	Dystric Regosol (Siltic)
P10	1.4	86.6	12.1	6.90	0.69	9.99	4.63	Dystric Regosol (Siltic)
P11	16	69.5	14.5	1.37	0.18	7.70	4.44	Dystric Regosol (Siltic)
P12	17	69.3	13.7	0.61	0.08	7.96	6.60	Eutric Regosol (Siltic)
P13	20	72.1	7.9	1.44	0.16	9.11	4.58	Dystric Regosol (Siltic)
P14	18	72.1	9.9	2.12	0.20	10.61	4.52	Dystric Regosol (Siltic)
P15	2.4	88.9	8.7	0.62	0.05	13.56	4.15	Dystric Regosol (Siltic)
P16	15	72	13.1	1.23	0.17	7.34	5.86	Eutric Regosol (Siltic)
P17	22	68.2	9.8	0.88	0.11	7.80	4.73	Dystric Regosol (Siltic)
P18	16	73.1	10.9	1.86	0.22	8.55	4.99	Dystric Regosol (Siltic)

Footnote: WRB 2022 soil names are assigned according to IUSS Working Group WRB. 2022, World Reference Base for Soil Resources (4th edition). TOC and TN are mass fractions calculated on oven-dry-soil basis, with hygroscopic-moisture correction.
